# Identification of a new way to induce differentiation of dermal fibroblasts into vascular endothelial cells

**DOI:** 10.1186/s13287-022-03185-4

**Published:** 2022-10-09

**Authors:** XiaoLing Cui, XiaoTan Wang, Jie Wen, Xiao Li, Nan Li, XuXiao Hao, BaoXiang Zhao, Xunwei Wu, JunYing Miao

**Affiliations:** 1 Shandong Provincial Key Laboratory of Animal Cells and Developmental Biology, School of Life Science, Shandong University, Qingdao, 266237 People’s Republic of China; 2 The First Clinical Medical School, Shandong University of Traditional Chinese Medicine, Jinan, 250014 People’s Republic of China; 3 Shandong Key Laboratory of Oral Tissue Regeneration and Shandong Engineering Laboratory for Dental Materials and Oral Tissue Regeneration, Department of Tissue Engineering and Regeneration, School and Hospital of Stomatology, Cheeloo College of Medicine, Shandong University, Jinan, People’s Republic of China; 4 Institute of Organic Chemistry, School of Chemistry and Chemical Engineering, Shandong University, Jinan, 250100 People’s Republic of China; 5 The Key Laboratory of Cardiovascular Remodeling and Function Research, Chinese Ministry of Education and Chinese Ministry of Health, Shandong University Qilu Hospital, Jinan, 250012 People’s Republic of China; 6 Engineering Laboratory for Biomaterials and Tissue Regeneration, Ningbo Stomatology Hospital, Savaid Stomatology School, Hangzhou Medical College, Ningbo, 315000 People’s Republic of China

**Keywords:** Small chemical molecule, Human dermal fibroblasts, Vascular endothelial cells, Differentiation

## Abstract

**Background:**

Human dermal fibroblasts (HDFs) have the potential to differentiate into vascular endothelial cells (VECs), but their differentiation rate is low and the mechanism involved is not clear. The small molecule pathway controls the phenotype of fibroblasts by activating cellular signaling pathways, which is a more convenient method in the differentiation strategy of HDFs into VECs.

**Methods:**

In this study, HDFs were treated with the different doses of CPP ((E)-4-(4-(4-(7-(diethylamino)-2-oxo-2H-chromene-3-carbonyl) piperazin-1-yl) styryl)-1-methylpyridin-1-ium iodide), and the mRNA and protein levels of HDFs were detected by qPCR, Western blot, flow cytometry and immunofluorescent staining. The matrigel assays, acetylated-LDL uptake and angiogenesis assays of chick embryo chorioallantoic membrane (CAM) and hindlimb ischemia model of nude mice were performed to evaluate the functions of VECs derived from HDFs.

**Results:**

Here, we report that the small chemical molecule, CPP, can effectively induce HDFs to differentiate into VECs. First, we observed the morphological changes of HDFS treated with CPP. Flow cytometry, Western blot and qRT-PCR analyses showed that CPP effectively decreased the level of the HDFs-marker Vimentin and increased levels of the VEC-markers CD31, CD133, TEK, ERG, vWF, KDR and CDH5. Detection of the percentage of CD31-positive cells by immunofluorescent staining confirmed that CPP can effectively induce HDFs to differentiate into VECs. The results of Matrigel assays, DiI-ac-LDL uptake, angiogenesis assays on CAM and hindlimb ischemia model of nude mice showed that CPP-induced HDFs have the functions of VECs in vitro and in vivo. Western blot and qRT-PCR analysis showed that CPP induces HDFs to differentiate into VECs by promoting the expression of pro-angiogenic factors (VEGF, FGF-2 and PDGF-BB).

**Conclusions:**

Our data suggest that the small chemical molecule CPP efficiently induces the differentiation of HDFs into VECs. Simultaneously, this new inducer provides a potential to develop new approaches to restore vascular function for the treatment of ischemic vascular diseases.

**Supplementary Information:**

The online version contains supplementary material available at 10.1186/s13287-022-03185-4.

## Background

The main reason for the damage of the vascular repair mechanism is vascular endothelial cells (VECs), that the endothelial progenitor cells in the body are damaged due to lack, poor mobilization or dysfunction [1, 2]. Therefore, many approaches have been developed to generate ECs for the use in cell therapy. However, the differentiation rate of using adult stem cells and progenitor cells to produce endothelial cells is relatively low [3, 4].

Human dermal fibroblasts (HDFs) in the skin are derived from mesenchymal stem cells (MSCs) during embryonic development [5, 6]. HDFs are abundant in the human body and have multi-directional differentiation potential. Previous studies have reported that HDFs can differentiate into endothelial-like cells, fat-like cells, cartilage-like cells, bone-like cells and spinal motor neurons [7–9]. At present, the main strategy to induce the differentiation of HDFs is transgenic technology. However, transgenic technology has the disadvantages of low differentiation rate and high cost, so it is urgent to develop more effective differentiation induction strategies [10–12].

The small molecule pathway can regulate the phenotype of fibroblasts by activating cellular stress-related signaling pathways, which provides a more convenient application method for the differentiation strategy of fibroblasts into endothelial cells. Also, small chemical molecules have been reported to possess great advantages in inducing cell differentiation, which can produce faster biological effects and contribute to the in-depth studies of signaling pathways [13, 14]. In our research, we are committed to using small chemical molecules as tools to discover new factors and new pathways [15–17]. Therefore, our aim is to identify new chemical molecules that can effectively induce HDFs to differentiate into endothelial cells. Recently, we synthesized and identified a new water-soluble fluorescence probe for the detection of hypochlorous acid (HOCl) ((E)-4-(4-(4-(7-(diethylamino)-2-oxo-2H-chromene-3-carbonyl) piperazin-1-yl)styryl)-1-methylpyridin-1-ium iodide)(CPP) [18], and observed the effects of CPP on the differentiation of HDFs.

## Methods and materials

### Animals

A total of 20 eight-week-old and pathogen‑free BALB/C nude mice with a mean weight of 20 g were housed at 20‑24˚C with 40–60% humidity and with a regular light–dark cycle. All animal experiments were performed according to Institutional Animal Care and Use Committee guidelines. All efforts were made to minimize animal suffering.

### Antibodies

Antibodies against CD31 (sc-1506), PDGF-BB (sc-7878), VEGF (sc-7269), FGF-2 (sc-271847) and CD133 (sc-30219) were from Santa Cruz Biotechnology (Santa Cruz, CA, USA). Antibodies against Vimentin (10,366–1-AP) were from Proteintech group (Wuhan, China). The antibody against β-actin was from Sigma-Aldrich (St. Louis, MO, USA). Horseradish peroxidase-conjugated secondary antibodies were from Jackson Immunoresearch (West Grove, PA, USA). The secondary antibody used for immunofluorescence was donkey anti-rabbit IgG Alexa Fluor-546 (A-11037; Invitrogen, Carlsbad, CA, USA).

### Cell culture

Human primary HDFs were derived from adult foreskins, and were isolated according to our previous publication [19]. HDFs were cultured in DMEM Basic medium (C11995500BT, Gibco, Grand Island, NY, USA) supplemented with 10% (v/v) bovine calf serum. HDFs were cultured in a humidified incubator at 37 °C in a 5% CO_2_ atmosphere. Cells were seeded in appropriate dishes (35,000 cells/ml), and all cell lines were authenticated by DNA short tandem repeat (STR) profiling and were confirmed to be mycoplasma negative.

### Cell morphology

Morphological changes of HDFs were examined using an inverted phase contrast microscope (Eclipse TS-100; Nikon, Tokyo, Japan) after 10 days of treatment with CPP at the indicated concentrations.

### Cell viability assay

HDFs were seeded in 96-well plates and were then treated with 0.1% DMSO (as a control) or with CPP at the indicated concentrations for 48 h. Cell viability was determined using a sulforhodamine B (SRB) assay (L109288, Aladdin, Shanghai, China) according to the manufacturer’s instructions.

### Western blot analysis

Cell lysates (30 μg protein per lane) were separated by SDS-PAGE, after which the proteins were transferred to polyvinylidene difluoride membranes. At room temperature, the membranes were blocked with 5% non-fat milk in TBST (TBS containing 0.05% Tween-20) for 1 h. After that, the membranes were incubated with the primary antibody at 4 °C overnight, then were washed with TBST three times for 5 min each. Each membrane was incubated with the secondary antibody at room temperature for 1 h, and then washed with TBST 3 times, for 5 min each time. Antibodies bound to proteins were detected using an enhanced chemiluminescence detection kit (34,080, Thermo Fisher, Waltham, MA, USA). Relative quantities of specific bands were analyzed by Image J software and were normalized to loading controls.

### Quantitative real-time PCR

RNA was extracted from the whole-cell fraction by the Trizol reagent method (Takara, Tokyo, Japan), and extracted total RNAs were reverse transcribed using the primer sequences of the target genes. Reverse transcription was performed using the PrimeScript RT reagent kit with gDNA Eraser (Takara). PCR reactions involved the use of SYBR Premix Ex Taq (Tli RNaseH Plus, Takara) and levels of expressed genes were measured by the 2^−ΔΔCt^ method with MxPro 4.00 (Stratagene, La Jolla, CA, USA). The following primers were used: VEGF: 5′-ATCGAGTACATCTTCAAGCCAT-3′ (forward) and 5′-GTGAGGTTTGATCCGCATAATC-3′ (reverse); FGF-2: 5′-CATCAAGCTACAACTTCAAGCA-3′ (forward) and 5′-CCGTAACACATTTAGAAGCCAG-3′ (reverse); PDGF-BB: 5′-ACCGCACCAACGCCAACTTC-3′ (forward) and 5′-TCTTCCGCACAATCTCGATCTTTCTC-3′ (reverse); CD31: 5′-TCAGACGTGCAGTACACGGA-3′ (forward) and 5′-CTTTCCACGGCATCAGGGAC-3′ (reverse); CD133: 5′-GTGGCGTGTGCGGCTATGAC-3′ (forward) and 5′-CCAACTCCAACCATGAGGAAGACG-3′ (reverse); Vimentin: 5′-GGTGGACCAGCTAACCAACG-3′ (forward) and 5′-TTGCAGGGTGTTTTCGGCTT-3′ (reverse); Actin: 5′-CCTGGCACCCAGCACAAT-3′ (forward) and 5′-GCCGATCCACACGGAGTACT-3′ (reverse); CDH5: 5′- AAAGAATCCATTGTGCAAGTCC-3′ (forward) and 5′-CGTGTTATCGTGATTATCCGTG-3′ (reverse); ERG: 5′-GGAGTGGGCGGTGAAAGAATATGG-3′ (forward) and 5′-GAGAAGGATGTCGGCGTTGTAGC-3′ (reverse); KDR: 5′-GGAGCTTAAGAATGCATCCTTG-3′ (forward) and 5′-GATGCTTTCCCCAATACTTGTC-3′ (reverse); TEK: 5′-CGTGATTGACACTGGACATAAC-3′ (forward) and 5′-GAGTTCATATTCTGTCCGAGGT-3′ (reverse); vWF: 5′-CCTGTTACTATGACGGTGAGAT-3′ (forward) and 5′-CATGAAGCCATCCTCACAGTAG-3′ (reverse).

### Matrigel assays

Aliquots of Matrigel were stored at − 80 °C and were melted in ice overnight immediately prior to use. After mixing the culture medium and Matrigel (3:1), 300 μl Matrigel was added to each well in 24-well plates. The 24-well plates were cultured in a humidified incubator at 37 °C in a 5% CO_2_ atmosphere for 30 min. Cells were digested with trypsin and were then resuspended in culture medium and seeded in the 24-well plates at a concentration of 4 × 10,000 cells/ml. Morphological changes of HDFs were observed using an inverted phase contrast microscope (Eclipse TS-100; Nikon). The lengths of renal tubules were analyzed by Image J software and were normalized to the control group.

### Immunofluorescence microscopy

HDFs were seeded onto confocal dishes (20 mm) (SPL, Korea) and treated with CPP for 10 days. Next, the cells were fixed in 4% paraformaldehyde for 20 min. After washing with 1 × PBS three times, permeated cells with 0.2% TritonX-100 for 2 min, then washed and blocked with donkey serum (1:30 dilution in 0.1 M PBS) at room temperature for 30 min. Cells were incubated with primary antibodies at 4℃ overnight. On the second day, the cells were washed with PBS three times, and then incubated with secondary antibodies (1:200) at 37 ℃ for 1 h. Fluorescence was detected by confocal fluorescence microscopy Zeiss LSM700 (Germany).

### Flow cytometry

HDFs were treated with 0.1% DMSO (as a control) or with CPP for 10 days. Next, cells were digested into single cells by 0.25% trypsin (Sangon Biotech) and collected into 15-mL centrifuge tubes. Centrifugation at 300 g for 15 min was performed, then the supernatant was discarded. Cells were washed twice with 1 × PBS and suspended in 1 × PBS supplemented with 2% (v/v) FBS by centrifugation at 300 g for 15 min each time. We discarded the supernatant. Cells were resuspended in 1 × PBS with 2% (v/v) FBS and incubated at 4℃for 1 h with antibodies as indicated below. After staining, the cells were analyzed on a flow cytometer (ImageStreamX MarkII, Merck, Billerica, MA, USA). Antibodies used included: Alexa Fluor® 488 anti-human KDR (VEGFR2) Antibody (359,914, Biolegend, San Diego, CA, USA); PE anti-human CDH5 (VE-Cadherin) Antibody (348,506, Biolegend, San Diego, CA, USA).

### Acetylated-LDL uptake assay

HDFs were treated with 0.1% DMSO (as a control) or with CPP for 10 days, cells were incubated with DiI-Ac-LDL (L3484, Invitrogen, Carlsbad, CA, USA) at 10 μg/ml in growth media for 4 h. Next, cells were fixed with 4% Paraformaldehyde (w/v) at room temperature for 20 min and washed with 1 × PBS three times. Finally, cells were rinsed and stained with DAPI and monitored by a laser scanning confocal microscope (Zeiss, Germany). Randomly select the field of view for cell count in each group (total cell count: about 200; The percentage of cells uptake Acetylated-LDL = (the cell counts of Acetylated-LDL uptake/the total cell counts) × 100%).

### Angiogenesis assay of chick embryo chorioallantoic membrane (CAM)

Fertilized chicken eggs were incubated at 37 °C with 60% relative humidity. HDFs were treated with 0.1% DMSO (as a control) or with CPP for 10 days, and then cells were digested into single cells by 0.25% trypsin (Sangon Biotech) and collected into 15-mL centrifuge tubes. Centrifugation at 1000 rpm for 8 min was performed. The supernatant was discarded, and the cells were incubated in the HBSS with 1.5 μM CM-DiI (C7000, Invitrogen, Carlsbad, CA, USA) for 5 min, and then for an additional 15 min at 4 ℃. After labeling, wash cells with phosphate-buffered saline (PBS). On embryonic day 7, eight million labeled HDFs or HUVECs in 20 μl of medium were seeded into chick embryo chorioallantoic membrane (CAM). After one week, 1 ml 4% paraformaldehyde was placed on the CAM and incubated for 30 min. Fluorescence was detected by the laser scanning confocal microscope Zeiss LSM900 (Germany). Randomly select the field of view, and calculate the number of cells around the blood vessel by ImageJ software.

### Hindlimb ischemia model

Hindlimb ischemia was generated in 8-week-old BALB/C nude mice as previously described [20, 21]. Briefly, the femoral artery was ligated, then it was cut off. Twenty nude mice with hindlimb ischemia were randomly divided into four groups, five in each group. HDFs were treated with 0.1% DMSO (as a control) or with CPP for 10 days, one million CPP-treated HDFs, DMSO-treated HDFs or HUVECs in 100 μl PBS or the same volume PBS without cells were intramuscularly injected into the ischemic hindlimb of BALB/C nude mice. Laser Doppler imaging was conducted to quantitatively measure hindlimb blood flow of nude mice using a PeriCam PSupporting Information System (Perimed AB, Sweden) every week up to the 4^th^ week. After sacrificed for tissue harvest, changes of muscle in the ischemic hindlimb of nude mice were assessed by histology analysis.

### Histological analysis

BALB/C nude mice were sacrificed at four weeks, hindlimb muscle tissues were fixed for 24 h in 4% paraformaldehyde, embedded in paraffin after dehydration, and cut into 5-μm slices. Hematoxylin and eosin-stained (H&E staining) paraffin sections were conducted to analyze muscle changes in the ischemic hindlimb of nude mice. For the calculation of necrotic area, the total area and necrotic area of H&E slices were calculated by Caseviewer software. Fixed and equilibrated tissues were embedded in Optimal Cutting Temperature (OCT) compound (Sakura Finetek USA, Torrance, CA, USA), snap-frozen overnight in -80 °C refrigerator, and cut at 10 μm. Frozen sections were incubated with fluorescein-labeled Griffonia (Bandeiraea) Simplicifolia Lectin I (BSL1, Vector Laboratory Inc.) at 37 °C to stain functional endothelial cells in blood vessels. Fluorescence was detected by the laser scanning confocal microscope Zeiss LSM900 (Germany).

### Statistical analysis

Data are reported as means ± SE from at least three independent experiments. Student’s *t*-test was performed to compare the mean between two groups. One-way ANOVA followed by multiple comparisons was used for comparison between more than two groups. For the hindlimb ischemia study, repeated measures ANOVA was used for comparison with LSD. Images were processed by GraphPad Prism 5 (GraphPad Software, USA) and Adobe Photoshop CC 2015 (Adobe, USA). Statistical significance was set at p < 0.05 using SPSS 17.0 (SPSS Inc., Chicago, IL, USA). For cell counts, randomly select the field of view for cell count in each group: the percentage of CD31-positive cells = (the count of CD31-positive cells/total cells) × 100%; The percentage of cells uptake Acetylated-LDL = (the cell count of Acetylated-LDL uptake/the total cell count) × 100%

## Results

### CPP altered the morphology of HDFs

**Fig. 1:**
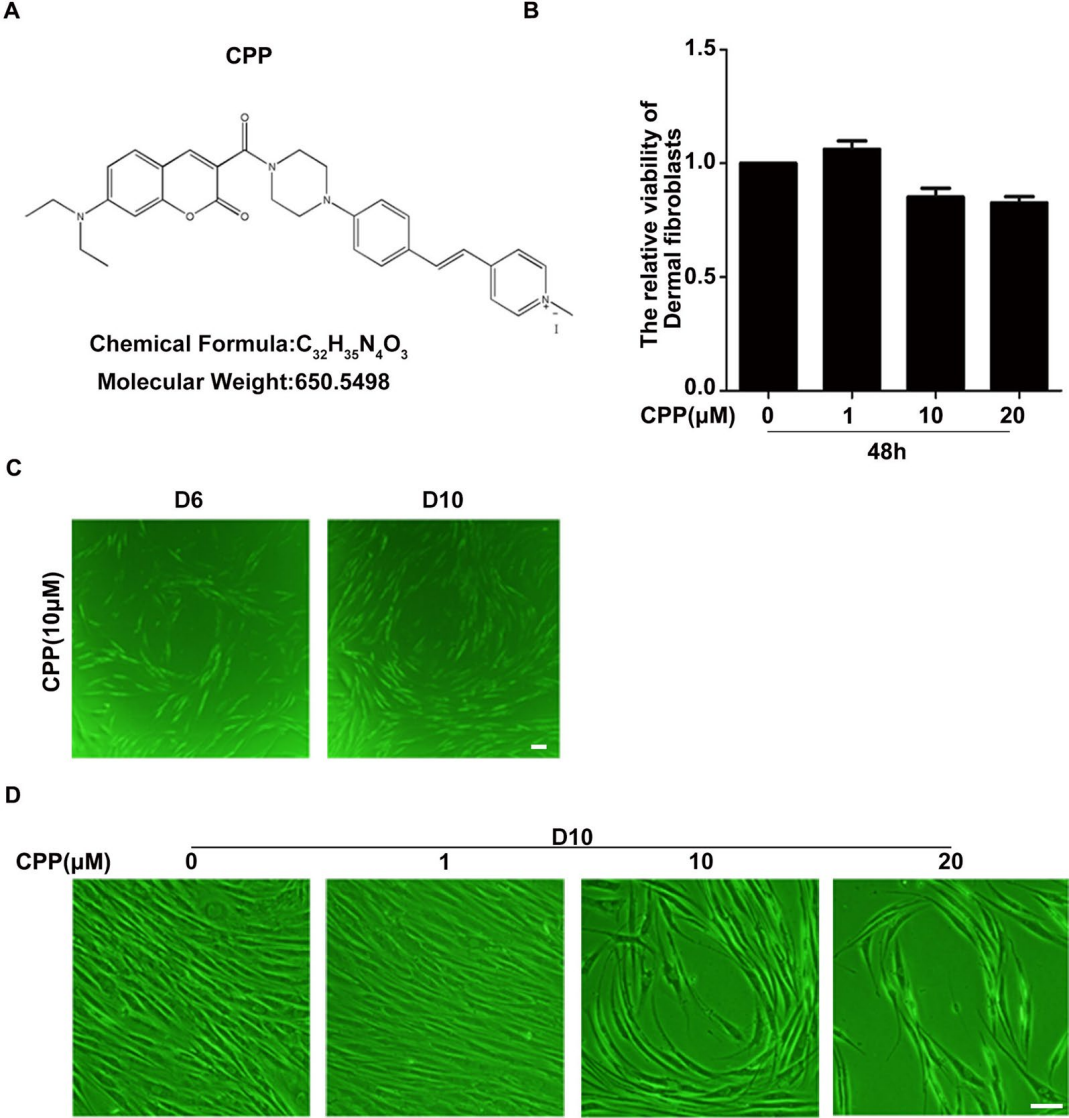
CPP induces morphological changes of HDFs. **A** The chemical structure of CPP. **B** Cell viability was determined using the sulforhodamine B (SRB) assay according to the manufacturer’s instructions. HDFs were seeded in 96-well plates and were then treated with 0.1% DMSO (as a control) or with CPP at the indicated concentration for 48 h, after which the SRB assay was used to determine cell viability. **C**,** D** HDFs were treated with the small chemical molecule CPP (0, 1, 10 or 20 μM) for 6 days (D6) or 10 days(D10), after which morphological changes of HDFs were examined using an inverted phase contrast microscope (Eclipse TS-100; Nikon, Tokyo). Scale bar: 20 μm

In order to test whether the hypochlorous acid (HOCl) probe CPP could potentially induce the differentiation of HDFs into endothelial cells, we first investigated whether CPP affects the cell viability of HDFs. Using the sulforhodamine B (SRB) assay, we observed that CPP had no significant effect on the viability of HDFs (Fig. 1A–B). Secondly, we investigated whether the treatment with CPP affected the morphology of HDFs. We observed morphological changes of HDFs treated with CPP for 6 and 10 days (Fig. 1C, D).

From the above results, we draw a conclusion that CPP, as a hypochlorous acid probe, affected the morphology of HDFs. To determine whether other hypochlorous acid probes also altered the morphology of HDFs, we treated HDFs with other HOCl probes for 10 days. Interestingly, other HOCl probes failed to alter the morphology of HDFs (Additional file 1: Fig. S1A). In conclusion, these data suggest that we have found a new inducer that can induce HDFs to change their morphology.

### CPP reduced the level of HDFs’ marker Vimentin

**Fig. 2:**
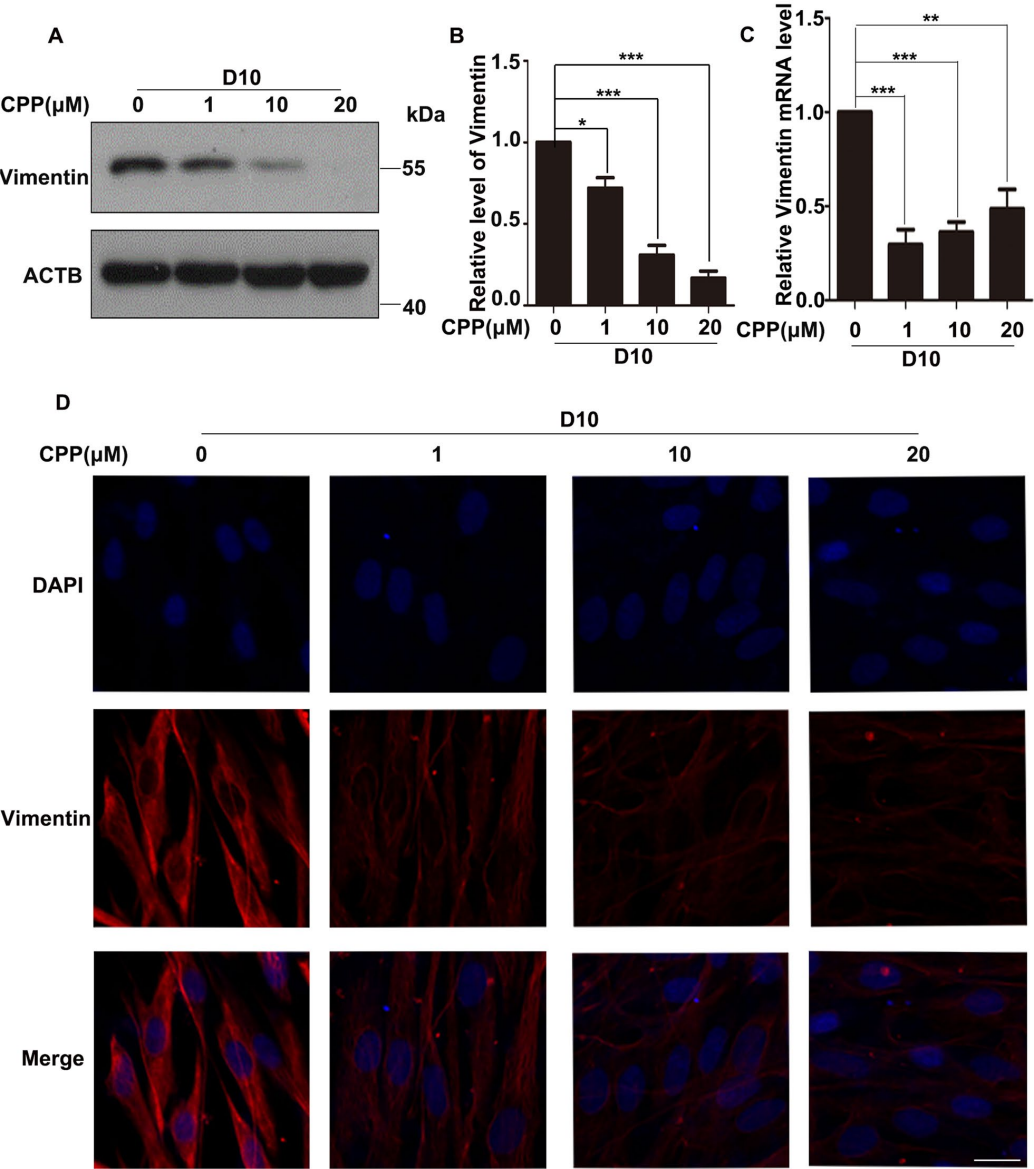
CPP reduced the expression of Vimentin. **A**–**B** HDFs were treated with 0, 1, 10 or 20 μM CPP for 10 days (D10), after which the protein level of Vimentin was determined by Western Blot. β-actin (ACTB) was used as a loading control. Quantitation of bands in the Western blots (**A**) is shown in (**B**). **C** Different doses of CPP (0, 1, 10 or 20 μM) were used to treat HDFs for 10 days, after which mRNA levels of Vimentin were detected by qPCR. **D** Different doses of CPP (0, 1, 10 or 20 μM) were used to treat HDFs for 10 days (D10), after which protein levels of Vimentin were detected by immunofluorescence. Scale bar: 20 μm. Data are presented as means ± SEM, *P < 0.05, **P < 0.01, *n* = 3

In the process of transforming one type of cell into another, the level of the marker protein in source cells will decrease, and the level of the marker protein in the target cell will increase. Therefore, in order to further prove that CPP induces HDFs to differentiate into VECs, we detected the marker protein Vimentin of HDFs. Western blot was used to detect the level of Vimentin in HDFs treated with CPP at 1, 10 and 20 μM for 10 days. We found that CPP significantly reduced the protein level of Vimentin at 10 μM (Fig. 2A–B). Interestingly, these results were verified by qRT-PCR analysis and by immunofluorescence staining (Fig. 2C–D).

### CPP promotes the expression of endothelial cell marker CD133

**Fig. 3:**
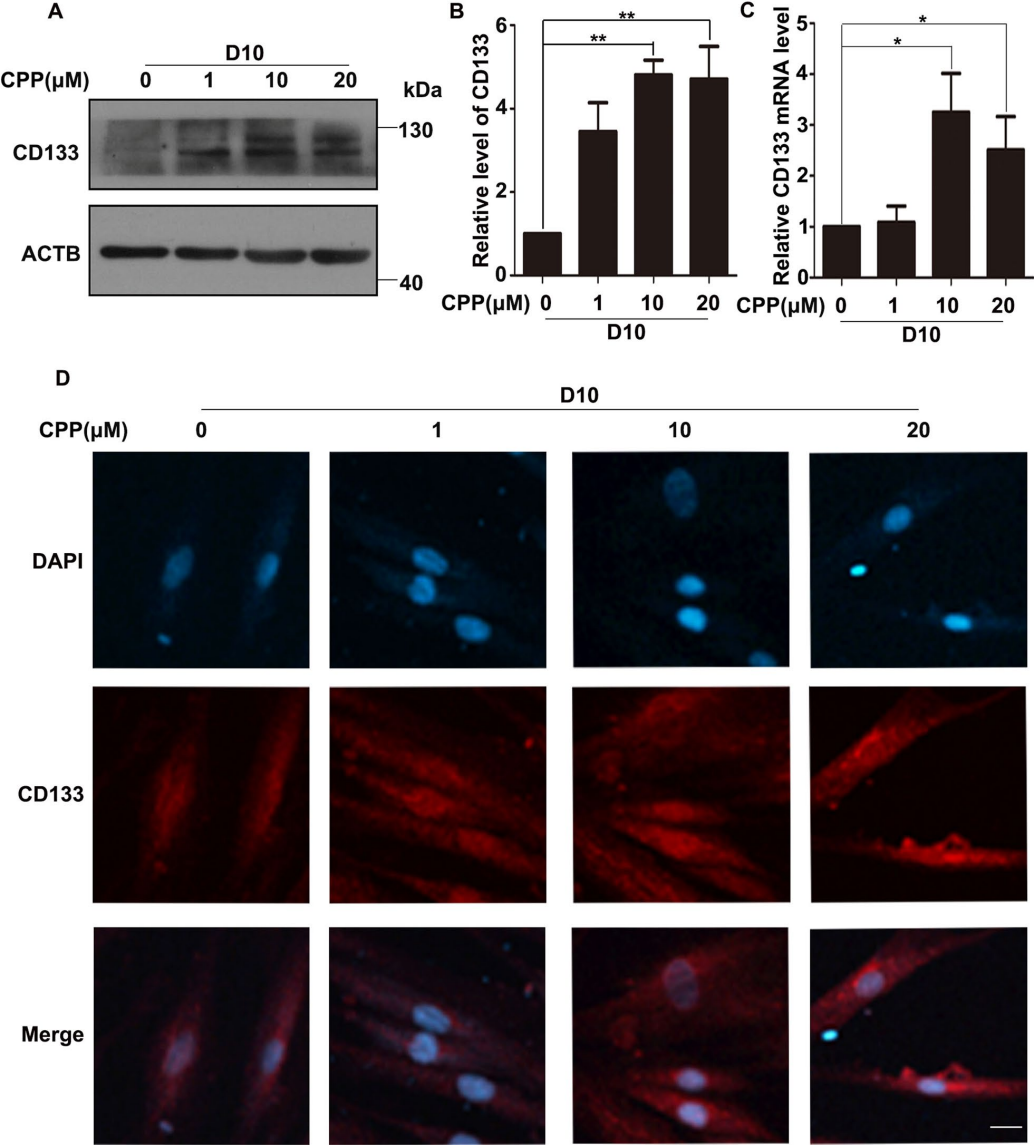
CPP promoted the expression of CD133. **A**–**B** HDFs were treated with 0, 1, 10 or 20 μM CPP for 10 days (D10), after which the protein level of CD133 was determined by Western Blot. β-actin (ACTB) was used as a loading control. Quantitation of bands in the Western blots (**A**) is shown in (**B**). (**C**) Different doses of CPP (0, 1, 10 or 20 μM) were used to treat HDFs for 10 days, after which mRNA levels of CD133 were detected by qPCR. (**D**) Different doses of CPP (0, 1, 10 or 20 μM) were used to treat HDFs for 10 days (D10), after which protein levels of CD133 were detected by immunofluorescence. Scale bar: 20 μm. Data are presented as means ± SEM, *P < 0.05, **P < 0.01, *n* = 3

In order to prove that HDFs differentiated into VECs after CPP treatment, we treated HDFs with different doses of CPP for 10 days. Cells treated with CPP had significantly increased protein levels of the endothelial cell marker CD133 (Fig. 3A, B). Next, qPCR analyses were conducted to detect the expression of CD133. Consistently, CPP could significantly increase the mRNA level of CD133 (Fig. 3C).

In addition, we used immunofluorescence staining to detect the level of CD133 in HDFs and found that CPP promoted the increase in CD133 level (Fig. 3D). Collectively, CD133, as an important regulator for the maintenance of endothelial progenitor cell stemness, plays an important role in the process of endothelial cell differentiation [22]. From the above data, we demonstrated that CPP significantly promoted the expression of CD133.

### CPP promotes the expression of endothelial cell marker CD31

**Fig. 4:**
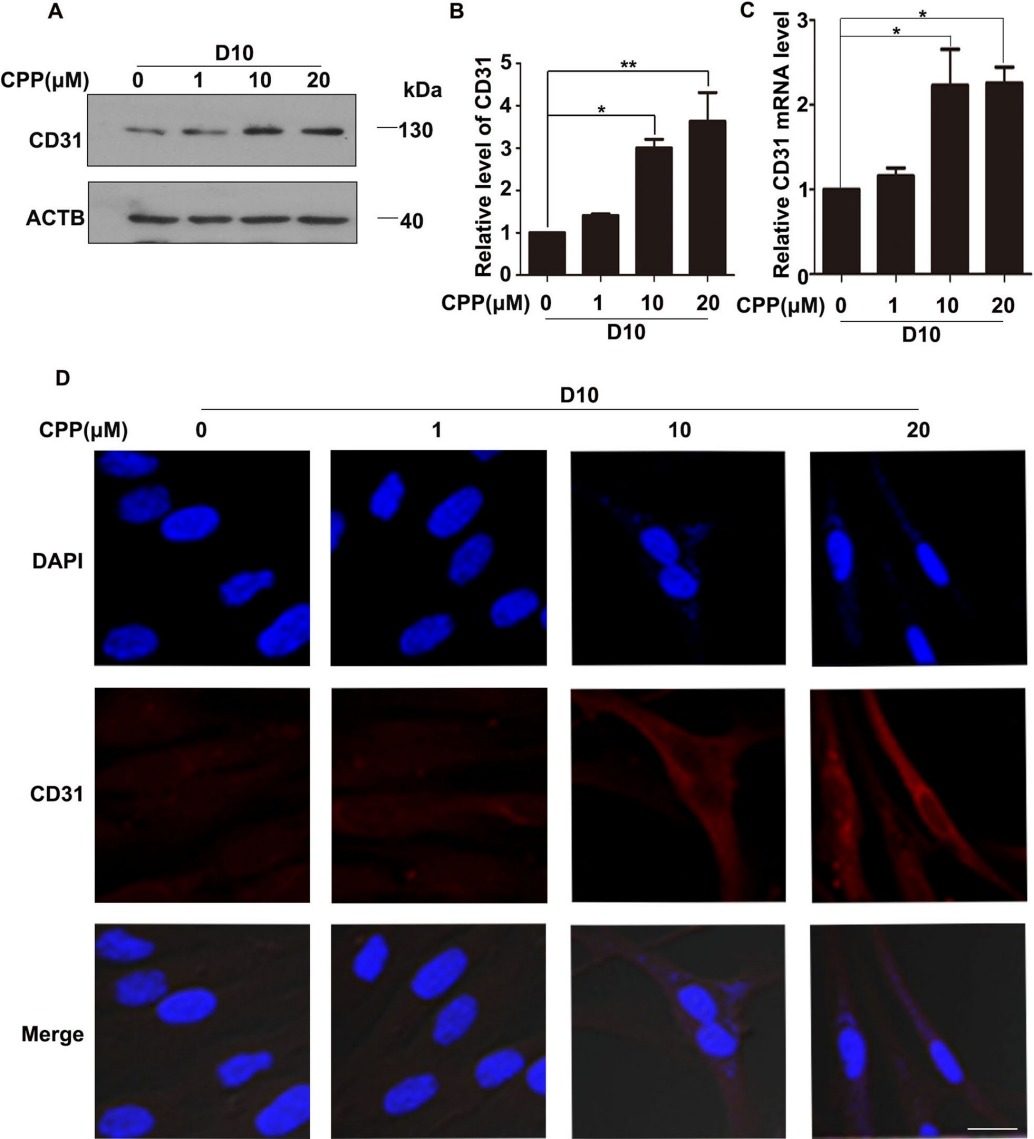
CPP promoted the expression of CD31. **A**–**B** HDFs were treated with 0, 1, 10 or 20 μM CPP for 10 days (D10), and the protein level of CD31 was determined by Western Blot. β-actin (ACTB) was used as a loading control. Quantitation of bands in the Western blots (**A**) is shown in (**B**). **C** Different doses of CPP (0, 1, 10 or 20 μM) were used to treat HDFs for 10 days, and the mRNA levels of CD31 were detected by qPCR. **D** Different doses of CPP (0, 1, 10 or 20 μM) were used to treat HDFs for 10 days (D10), after which protein level of CD31 was detected by immunofluorescence. Scale bar: 20 μm. Data are presented as means ± SEM, *P < 0.05, **P < 0.01, ***P < 0.001, *n* = 3

In order to prove that CPP induces HDFs to differentiate into VECs. We treated HDFs with CPP at 1, 10 and 20 μM for 10 days, the protein level of CD31 was measured by Western blot. As expected, the protein level of CD31 was significantly increased (Fig. 4A–B). Next, we detected the mRNA level of CD31 by qPCR, we also proved that the mRNA level of CD31 was increased (Fig. 4C). These results were verified by immunofluorescence staining (Fig. 4D). Together, these data suggested that CPP promoted the expression of CD31.

**Fig. 5:**
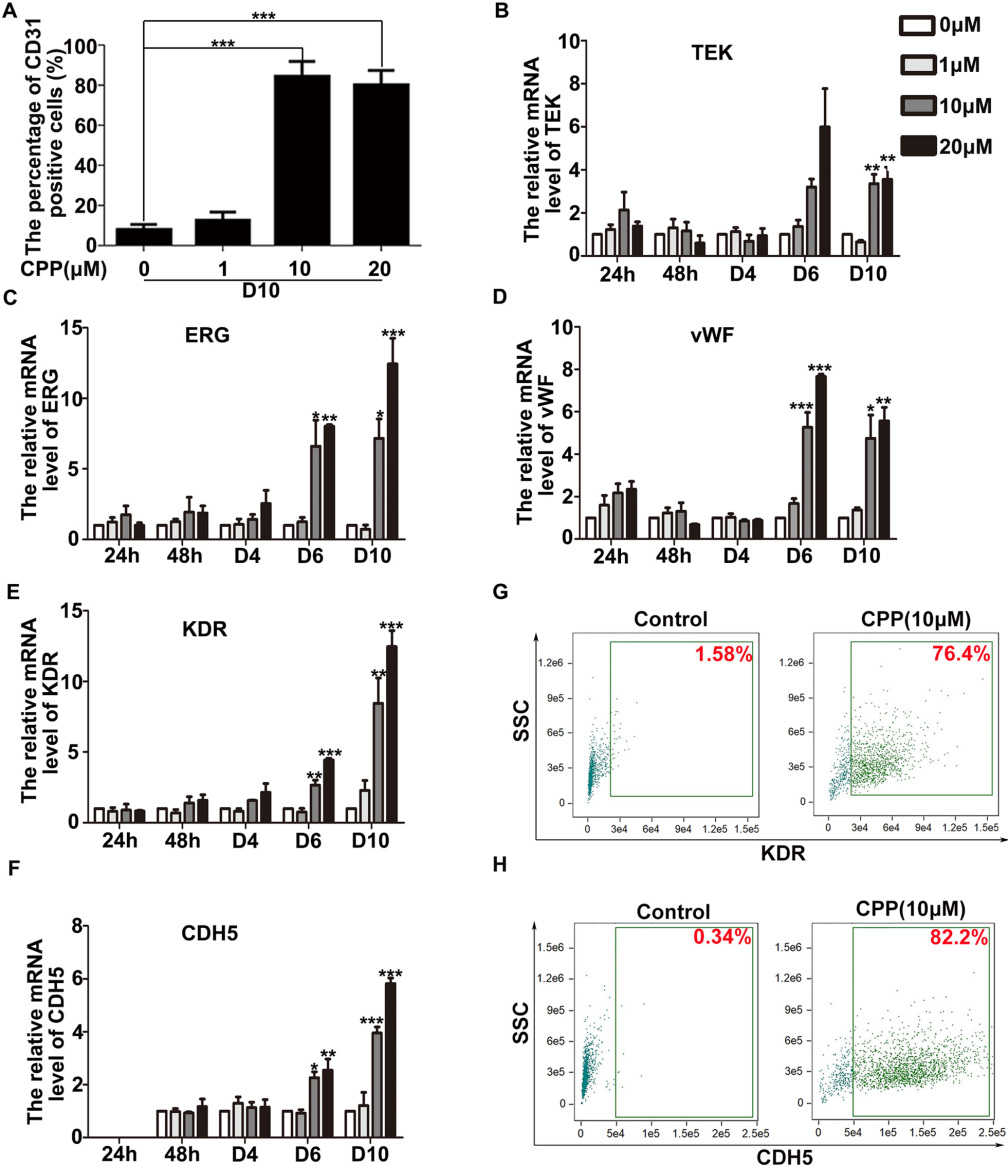
The differentiation rate and the expression of VEC genes. **A** Different doses of CPP (0, 1, 10 or 20 μM) were used to treat HDFs for 10 days, and then the ratio of the number of CD31-positive cells to the total number of cells was counted. Different doses of CPP (0, 1, 10 or 20 μM) were used to treat HDFs for 10 days, and treated HDFs expressed VEC genes and proteins measured by qRT-PCR (**B**–**F**) and flow cytometry (**G**–**H**), respectively. Data are presented as means ± SEM, *P < 0.05, **P < 0.01, ***P < 0.001, *n* = 3

Moreover, we quantified the percentage of CD31-positive cells using immunofluorescence staining, and found that nearly 80% of cells after 10 days of treatment with 10 or 20 μM CPP expressed CD31, and less than 10% of CD31-positive cells were observed in the control group (Fig. 5A). Taken together, we demonstrated that CPP effectively induced the differentiation of HDFs into VECs in vitro.

### CPP promotes the expression of VEC genes, ERG, vWF, KDR and CDH5

In order to prove that HDFs indeed differentiated into VECs after CPP treatment, we treated HDFs with different doses of CPP for different times and conducted qRT-PCR experiments. The mRNA levels of VEC genes ERG, vWF, KDR and CDH5, but not TEK, were markedly increased compared to the control group at D6. Interestingly, after 10 days, the mRNA levels of the five VEC genes all increased significantly (Fig. 5B–F). The results of flow cytometry confirmed the protein levels of VEC genes at D10, showing that approximately 75–85% of the cells expressed KDR or CDH5 (Fig. 5G–H). Taken together, these data indicated that CPP was able to induce VEC characteristics in HDFs.

### The VECs derived from HDFs have the capability for vessel formation in vitro and in vivo

**Fig. 6:**
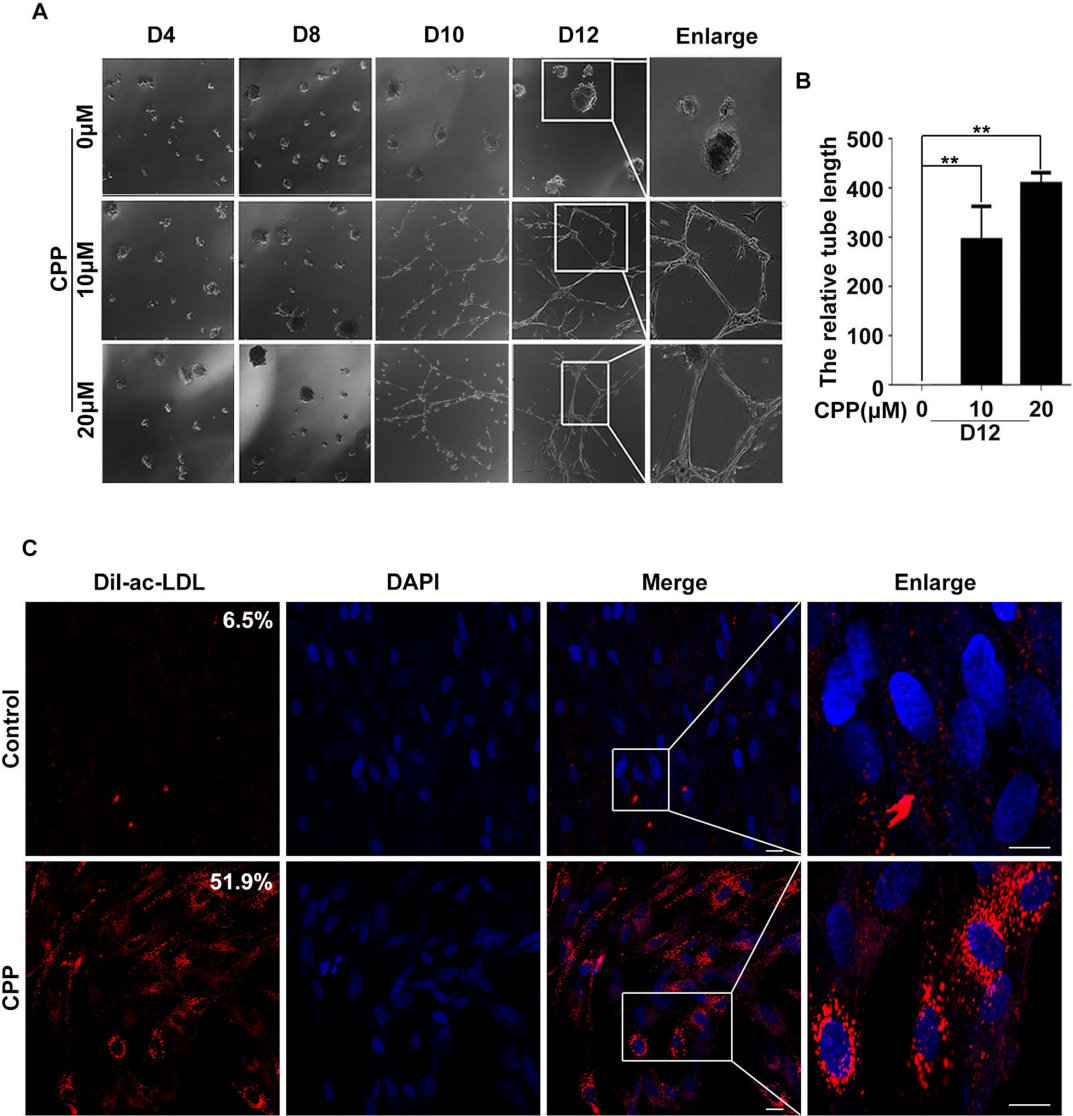
VECs derived from HDFs have the functions of VECs in vitro. **A**–**B** In vitro capillary-like tube formation by HDFs treated with different concentrations of CPP (0, 10, 20 μM) for 4, 8, 10 and 12 days. Representative images of capillary morphogenesis are shown at different points in time (**A**), and tubular length at day 12 (D12) was analyzed by ImageJ software and normalized to the control group (**B**). **C** HDFs were treated with or without CPP for 10 days (D10), the laser scanning confocal microscope Zeiss LSM900 (Germany) was used to observe LDL uptake. The ratio of DiI positive cell = (The number of DiI positive cells/The number of cells) × 100%. Scale bar: 20 μm. Data are presented as means ± SEM, **P < 0.01, *n* = 3

Next, we tested the functions of VECs derived from HDFs using an in vitro Matrigel tube formation assay. VECs derived from HDFs formed tube-like structures that didn’t appear in the control group without treatment of CPP (Fig. 6A, B).

In order to further verify the function of VECs derived from HDFs, we conducted the experiments of acetylated-LDL uptake. The result showed that approximately 51.9% of CPP-treated HDFs took up Ac-LDL (Fig. 6C).

**Fig. 7:**
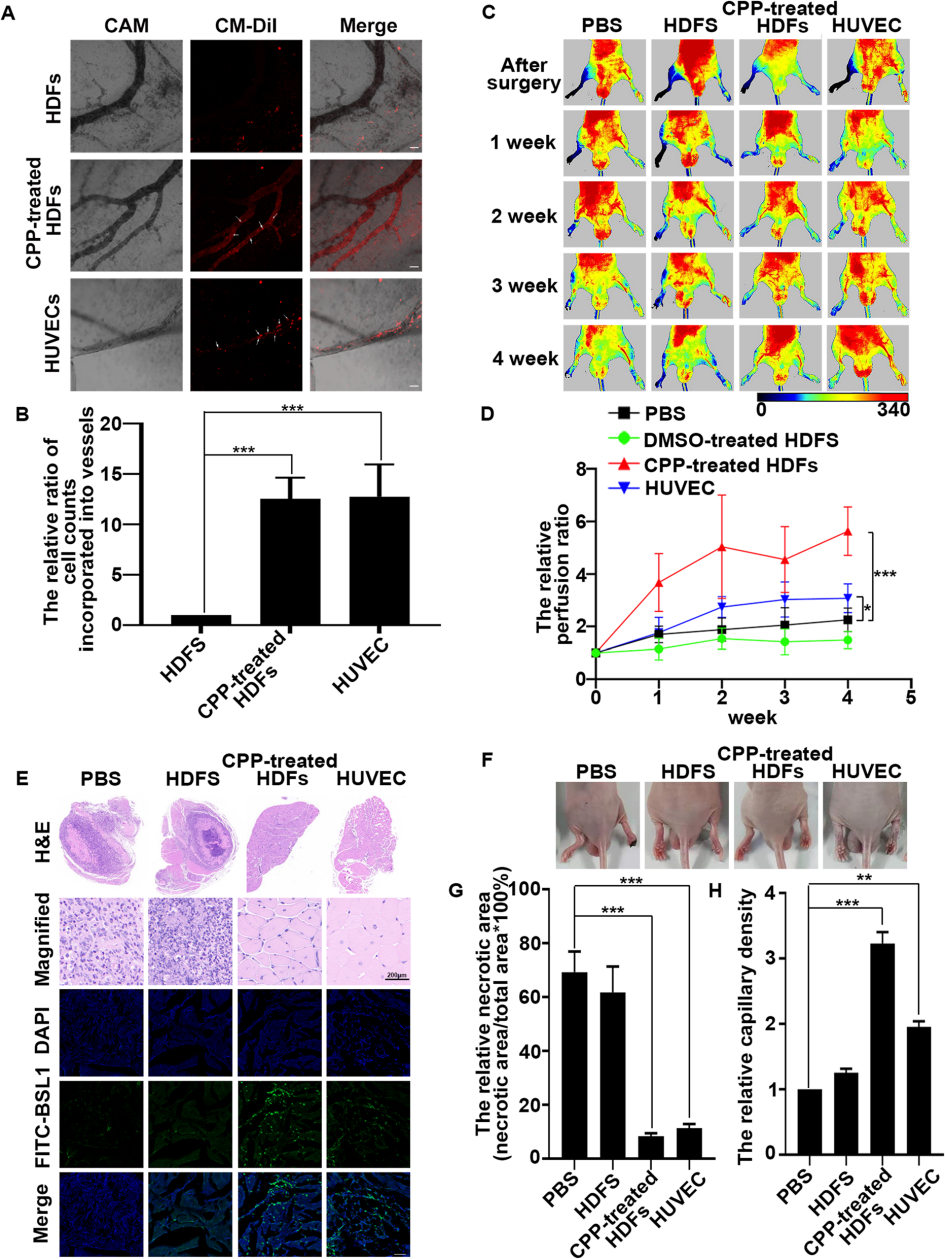
VECs derived from HDFs have the function of angiogenesis in vivo*.* **A** HDFs were treated with 0.1% DMSO (as a control) or with CPP for 10 days, and then treated cells and HUVECs were labeled by CM-DiI. Labeled cells were seeded into CAM. One week later, the fluorescence was detected by the laser scanning confocal microscope Zeiss LSM900 (Germany). Scale bar: 50 μm. **B** The number of cells incorporated into vessels was counted by ImageJ. One million CPP-treated HDFs, DMSO-treated HDFs or HUVECs in 100 μl PBS or the same volume PBS without cells were intramuscularly injected into the ischemic hindlimb of BALB/C nude mice, Laser Doppler perfusion images (**C**) and quantitative analysis of blood flow **D** showed improved limb perfusion, Data are presented as means ± SEM, *P < 0.05, **P < 0.01, ***P < 0.001, *n* = 5. The representative pictures of every group (**F**), **E** and **H** staining of paraffin sections and the fluorescence intensity of FITC-BSL1 (**E**) at 28 day showed the change of hindlimb and the number of functional endothelial cells. The necrotic area (**G**) and the Capillary density (**H**) is quantified by H&E staining and FITC-BSL1 staining. Scale bar: 200 μm (**H** and **E**); 50 μm (FITC-BSL1). Data are presented as means ± SEM, *P < 0.05, **P < 0.01, ***P < 0.001, *n* = 3

We then evaluated vessel-forming capability in vivo using CAM. DMSO- or CPP-treated HDFs were labeled and seeded into CAM. One week later, laser scanning confocal microscopy examination of the CAM showed that the CPP-treated HDFs were either incorporated into vessels or localized in close proximity to the vessels, indicating the contribution of VECs derived from HDFs to vessel formation in vivo. Consistently, the phenomenon was also observed in CAM seeded with HUVECs, but not in the control group (Fig. 7A, B).

### The transplantation of HDFs-derived VECs enhanced recovery from limb ischemia and increased the number of capillaries

The HDF-derived VECs exhibited functional EC characteristics both in vitro and in vivo assays as described above. In order to investigate the therapeutic effects of HDF-derived VECs on tissue ischemia, we intramuscularly injected CPP-treated HDFs into hindlimbs of the nude mice. Laser Doppler imaging was conducted to quantitatively measure hindlimb blood flow of nude mice. The results revealed significantly enhanced blood perfusion in the CPP-treated HDFs-injected limbs compared to the HUVEC-, DMSO-treated HDFs or phosphate-buffered saline (PBS)-injected limbs at 1, 2, 3, and 4 weeks (Fig. 7C, D). In H&E-stained cross sections of the calf (gastrocnemius) muscle, Muscle sections of the hind limbs showed severe necrosis in mice received DMSO-treated HDFs or PBS. However, in the mice received CPP-induced HDFS or HUVEC, the situation improved and the intact muscle structure basically covered the entire section areas (Fig. 7E, G). Consistently, mice received CPP-treated HDFs indicated better hindlimb repair compared to those received HUVEC, DMSO-treated HDFs or PBS (Fig. 7F). Next, FITC-BSL1 can be used to stain functional endothelial cells in the muscles of ischemic hindlimbs in mice to characterize the density of capillaries. Therefore, the frozen-sections were stained by FITC-BSL1, and the capillary density in the hindlimb muscle was significantly higher in those mice injected with CPP-treated HDFs than in the mice injected with the HUVEC, DMSO-treated HDFs- or PBS at day 28 (Fig. 7E, H). Collectively, these data suggested that VECs derived from HDFs possessed the function of VECs in vitro and in vivo. In addition, we also proved that VECs derived from the differentiation of HDFs induced by CPP were able to treat hindlimb ischemia in mice.

### CPP induces HDFs to differentiate into VECs through promoting the expression of pro-angiogenic factors

**Fig. 8:**
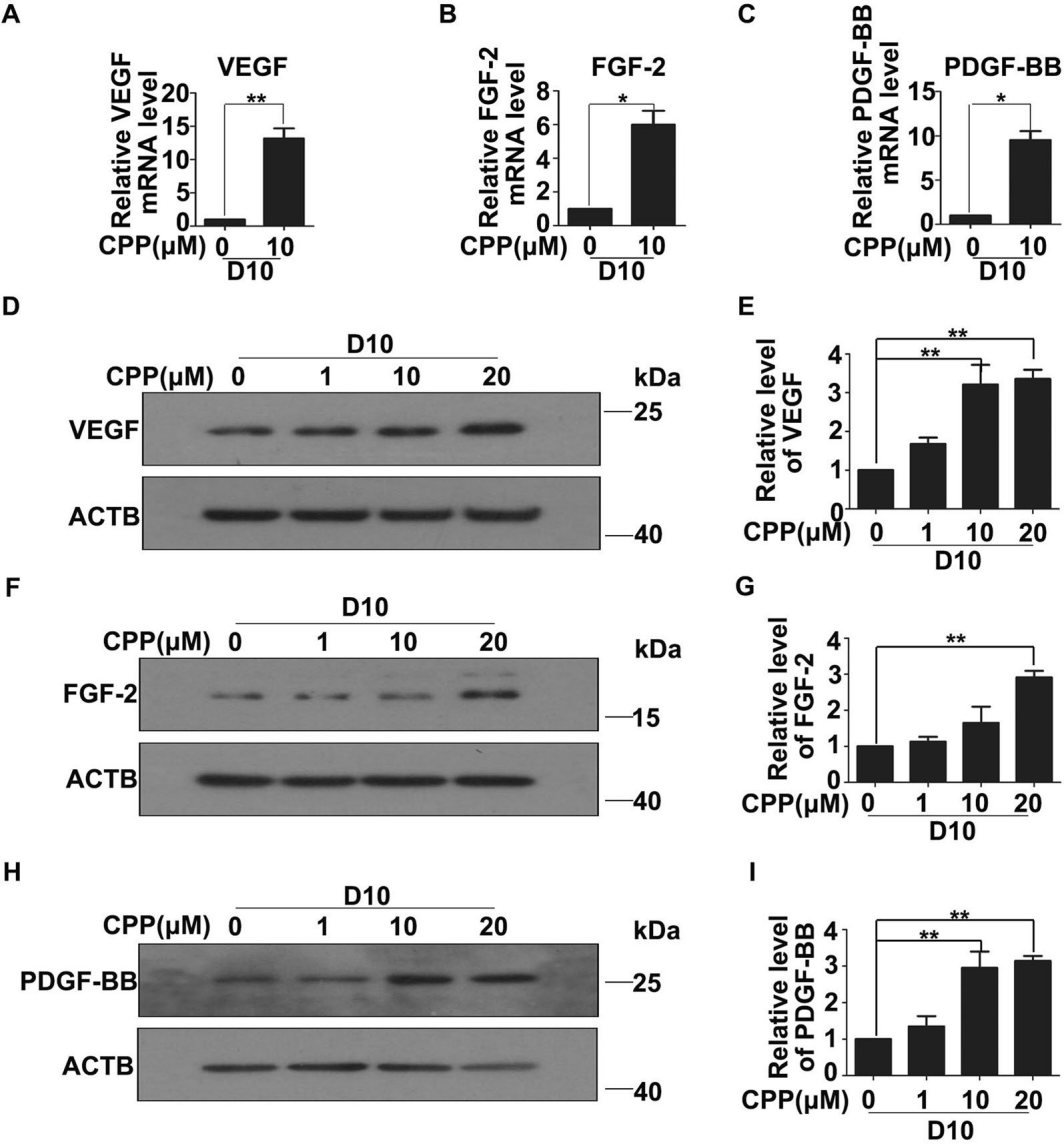
HDFs treated with CPP secrete angiogenesis-related factors. **A**–**C** mRNA expression levels of VEGF, FGF-2 and PDGF-BB in HDFs treated with or without CPP (10 μM) for 10 days (D10) were analyzed by qPCR. (D)-(I) HDFs were treated with or without CPP for 10 days (D10), and protein levels of VEGF, FGF-2 and PDGF-BB were determined by Western Blot (**D**), (**F**) and (**H**). β-actin (ACTB) was used as a loading control. Quantitation of bands in the Western blot bands (**D**), (**F**) and (**H**) are shown in (**E**), (**G**) and (**I**). Data are presented as means ± SEM, *P < 0.05, **P < 0.01, ***P < 0.001, *n* = 3

As a secretory cell, VECs can secrete a variety of cytokines. Studies have shown that Vascular endothelial growth factor (VEGF), fibroblast growth factor 2 (FGF-2) and Platelet-derived growth factor (PDGF-BB) are closely related to the maturation of VECs. In order to clarify the mechanism of CPP-induced differentiation of HDFs into VECs, we analyzed whether CPP(0,1,10 and 20 μM) treatment enhanced the expression levels of vascular endothelial function related factors in HDFs, and qRT-PCR analysis revealed that mRNA levels of VEGF, FGF-2 and PDGF-BB were strongly increased in the cells treated with CPP for 10 days (Fig. 8A–C), which were further confirmed by Western blot analysis (Fig. 8D–I). These data suggested that CPP induced HDFs to differentiate into VECs through promoting the expression of pro-angiogenic factors.

## Discussion

HDFs have the potential to differentiate into VECs, but at present, there are few effective strategies to induce HDFs to differentiate into VECs. Lee et al. demonstrated the direct reprogramming of human HDFs into endothelial cells using ER71/ETV2 [21]. Recently, analyses of induced neuron production by single cell RNA-Seq revealed that silencing of reprogramming factors, death from an epigenetically unstable state and reprogramming toward alternative fates, limited the number of cells successfully reprogrammed [23, 24]. Therefore, there are some safety issues in the genetic delivery of exogenous genes, such as gene mutations or insertions, etc. [25–27]. In this work, based on a simple, efficient and economical induction method provided by chemical small molecules, CPP was found to be able to induce HDFs to differentiate into VECs with a high rate of differentiation.

More and more evidence shows that small chemical molecules can regulate cell phenotypes by targeting signaling pathways, epigenetic modifications and metabolic processes [28, 29]. In the study of signaling pathways, it was found that the Wnt signaling pathway, the TGF-β signaling pathway and the MAPK/ERK signaling pathway play important roles in the maintenance of cell pluripotency [30–32]. Small molecules maintain cell pluripotency by affecting those signaling pathways [33]. However, in previous studies, researchers used a variety of small molecules to treat cells together to achieve a high induction rate [28, 34, 35]. Since different small chemical molecules regulate physiological processes by different signal pathways, it is difficult to study the mechanism of cell differentiation when treating cells with a group of various small molecules. In this study, we just used a small chemical molecule CPP to induce differentiation of HDFs into VECs with the differentiation rate up to 80%. Furthermore, we also proved that the VECs derived from HDFs have the capability for vessel formation in vitro and in vivo.

The CAM experiment indirectly proved that the VECs derived from the differentiation of HDFs induced by CPP have the ability to participate in angiogenesis. In order to further evaluate the function of CPP-treated HDFS in vivo, we used a hindlimb ischemia model of mice to conduct experiments and found that mice injected with CPP-treated HDFs could restore blood flow and repair the muscle tissue of the hind limbs to the greatest extent. Studies have found that severe limb ischemia secondary to peripheral arterial disease is a disabling and potentially fatal disease. In many cases, surgery or catheter-based revascularization is impossible and requires amputation. For these reasons, the development of molecular or cellular therapies to promote angiogenesis continues to be a major area of scientific and clinical interest. Therefore, we not only use the hindlimb ischemia model to prove that the VECs produced by CPP-induced HDFS have functions in vivo, but also provide a potential therapy for the treatment of hindlimb ischemic diseases.

VEGF regulates the function of endothelial cells through its three receptors VEGFR1, VEGFR2, and VEGFR3 [36]. At the same time, the VEGF signaling pathway plays a regulatory role in the differentiation of endothelial progenitor cells into VECs [37, 38]. In this study, we found that the expression of VEGF in the CPP-treated HDFs was significantly increased, suggesting that VEGF played a notable role in the differentiation of HDFs into VECs. As is known, PDGF signaling pathway also plays an important role in the differentiation of endothelial progenitor cells into mature endothelial cells [39, 40], and PDGF-BB can induce the production of mature endothelial cells under serum-free conditions. In a previous study, it was found that VEGF and FGF-2 synergistically activate the endogenous PDGF-B-PDGFRβ signaling pathway [41]. Therefore, in this study, compared with the control group, HDFs treated with CPP significantly increased the levels of VEGF, FGF-2 and PDGF-BB.

## Conclusions

In conclusion, we have found a new induction method that can induce the differentiation of HDFs into VECs. These results provide new ideas for the mechanism of study of HDF differentiation, and also provide a new compound to potentially develop effective new drugs against ischemic dermopathy.

## Supplementary Information

**Additional file 1**. Fig. S1. **A** HDFs were treated with other HOCI probes for 10 days, and morphological changes of HDFs were observed under an inverted phase contrast microscope (Eclipse TS-100; Nikon, Tokyo). Scale bar: 20 μm.

## Data Availability

The data generated or analyzed during this study are included in this article, or if absent are available from the corresponding author upon reasonable request.

## Abbreviations

MSCs: Mesenchymal stem cells
HOCl: Hypochlorous acid
HDFs: Human dermal fibroblasts
VECs: Vascular endothelial cells
VEGF: Vascular endothelial growth factor
FGF-2: Fibroblast growth factor 2
PDGF: Platelet-derived growth factor
