# Supplementary figures and images for: Identification of a new way to induce differentiation of dermal fibroblasts into vascular endothelial cells
Source: Stem Cell Res Ther. 2022 Oct 9;13:501. doi: 10.1186/s13287-022-03185-4 (PMC9549676; doi:10.1186/s13287-022-03185-4)

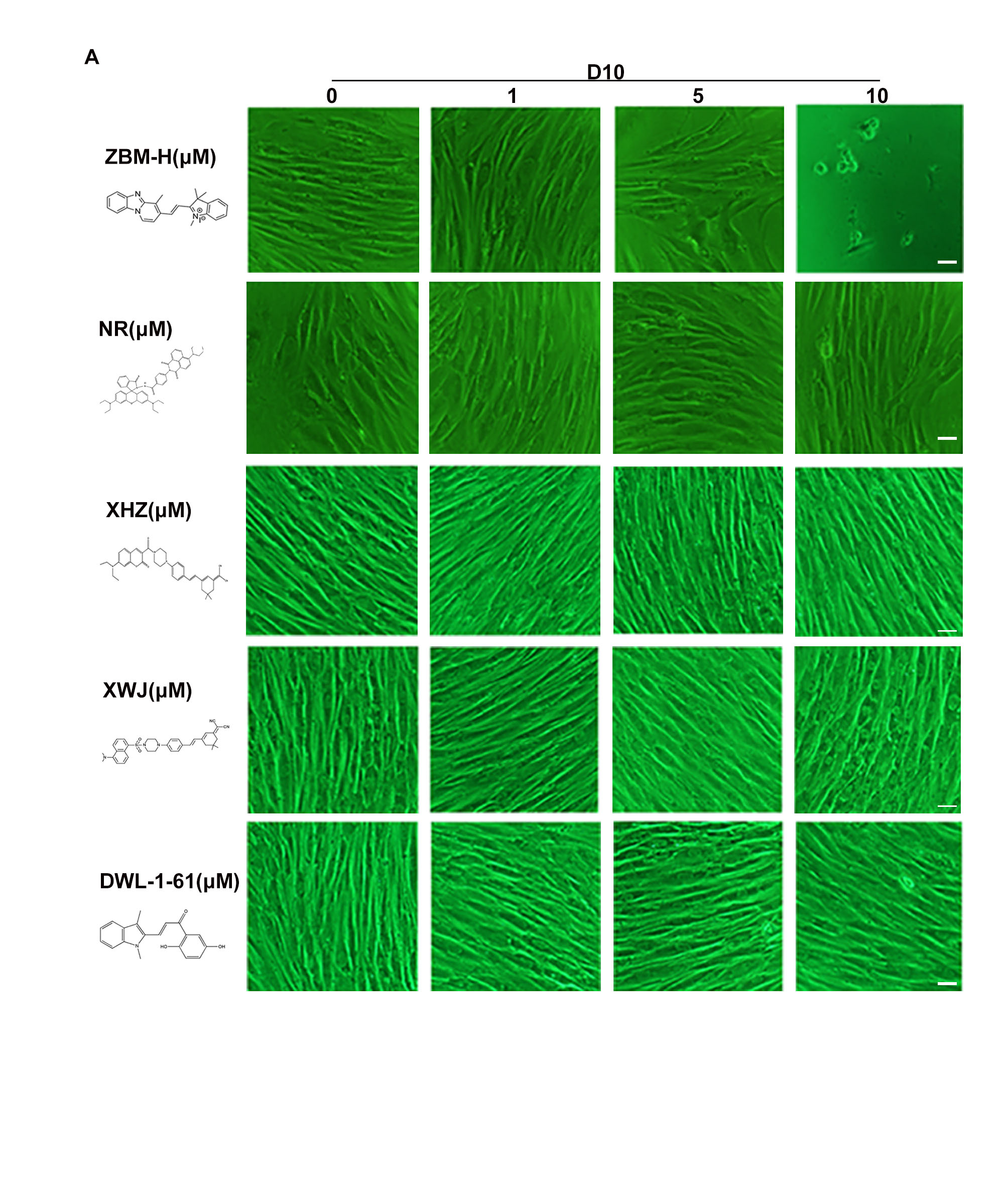

Supplement: Supplementary file 1 — Additional file 1. Fig. S1. A HDFs were treated with other HOCI probes for 10 days, and morphological changes of HDFs were observed under an inverted phase contrast microscope (Eclipse TS-100; Nikon, Tokyo). Scale bar: 20 μm. [file 13287_2022_3185_MOESM1_ESM.jpg]
